# 4α,6α-Dihy­droxy-1β-methyl­sulfonyl-8α,9α-ep­oxy-2β,12-epoxymethano-β-dihydro­agarofuran

**DOI:** 10.1107/S1600536811034179

**Published:** 2011-08-27

**Authors:** Jiwen Zhang, Peng Gao, Longbo Li, Wenjun Wu

**Affiliations:** a Institute of Pesticide Science and College of Sciences, Northwest A&F University, Yangling 712100, Shannxi Province, People’s Republic of China; b College of Science, Northwest A&F University, Yangling 712100, Shannxi Province, People’s Republic of China

## Abstract

The title mol­ecule, C_16_H_24_O_8_S, is a dihydro­agrofuran derivative and has a heteropolycyclic structure. One cyclohexane ring exhibits a chair conformation and the other a non-chair conformation. In the crystal structure there is an inter­molecular C—H⋯O hydrogen-bonding inter­action to stabilize the packing.

## Related literature

For general background, see: Gao *et al.* (2007 ▶); Spivey *et al.* (2002 ▶).
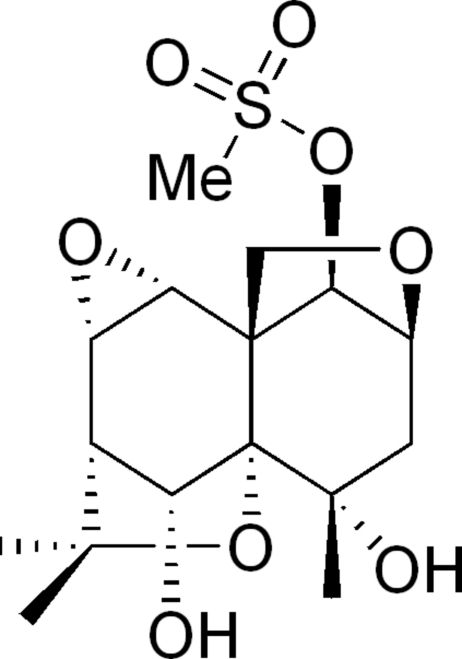


## Experimental

### 

#### Crystal data

C_16_H_24_O_8_S
*M* *_r_* = 376.41
Orthorhombic, 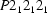

*a* = 9.530 (3) Å
*b* = 10.228 (3) Å
*c* = 17.424 (5) Å
*V* = 1698.3 (9) Å^3^
*Z* = 4
Mo *K*α radiation
μ = 0.23 mm^−1^
*T* = 296 K
0.25 × 0.22 × 0.21 mm

#### Data collection

Bruker APEXII CCD diffractometer
Absorption correction: multi-scan (*SADABS*; Sheldrick, 1996 ▶) *T*_min_ = 0.944, *T*_max_ = 0.953
8175 measured reflections
3151 independent reflections
2633 reflections with *I* > 2σ(*I*)
*R*_int_ = 0.034

#### Refinement

*R*[*F* ^2^ > 2σ(*F* ^2^)] = 0.039
*wR*(*F* ^2^) = 0.085
*S* = 1.06
3151 reflections
232 parameters
H-atom parameters constrained
Δρ_max_ = 0.19 e Å^−3^
Δρ_min_ = −0.20 e Å^−3^
Absolute structure: Flack (1983 ▶), 1325 Friedel pairs
Flack parameter: 0.13 (9)

### 

Data collection: *APEX2* (Bruker, 2005 ▶); cell refinement: *SAINT* (Bruker, 2001 ▶); data reduction: *SAINT*; program(s) used to solve structure: *SHELXS97* (Sheldrick, 2008 ▶); program(s) used to refine structure: *SHELXL97* (Sheldrick, 2008 ▶); molecular graphics: *SHELXTL* (Sheldrick, 2008 ▶); software used to prepare material for publication: *SHELXTL*.

## Supplementary Material

Crystal structure: contains datablock(s) global, I. DOI: 10.1107/S1600536811034179/qm2023sup1.cif

Structure factors: contains datablock(s) I. DOI: 10.1107/S1600536811034179/qm2023Isup2.hkl

Additional supplementary materials:  crystallographic information; 3D view; checkCIF report

## Figures and Tables

**Table 1:** Hydrogen-bond geometry (Å, °)

*D*—H⋯*A*	*D*—H	H⋯*A*	*D*⋯*A*	*D*—H⋯*A*
C16—H16*C*⋯O2^i^	0.96	2.32	3.218 (4)	156

Symmetry code: (i) 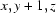.

**Fig. 1.**
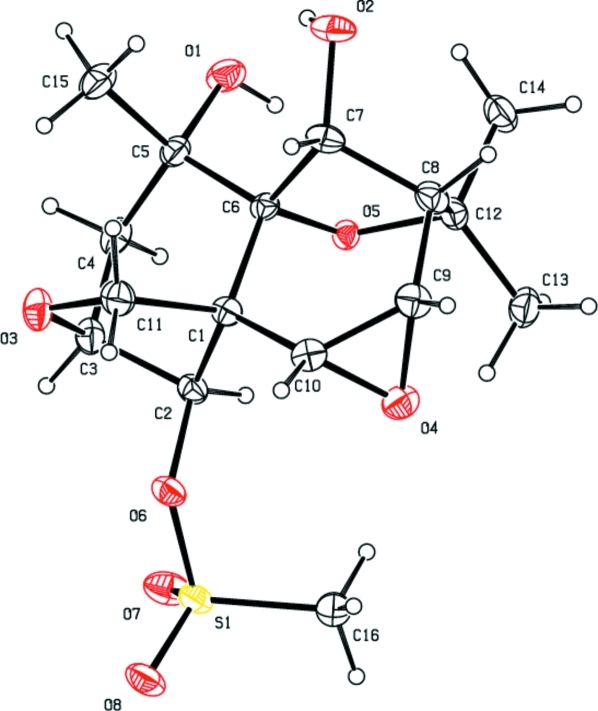
The molecular structure of the title compound with the atom numbering scheme. Displacement ellipsoids are drawn at the 30% probability level.

## supplementary crystallographic information

### Comment

Dihydroagrofuran esters and their derivatives have been widely explored as insecticidal medicine (Gao *et al.* 2007; Spivey *et al.* 2002). As a result of our program of screening insecticidal activity constituents from plants of China, some insecticidal β-dihydroagrofuran sesquiterpene polyol esters were isolated from the root bark of Chinese bittersweet, *Celastrus angulatus* Max. In order to find a new synthetic insecticide, the /b-dihydroagrofuran sesquiterpene polyol ester was optimized as a lead compound and we obtained an intermediate compound C_16_H_24_O_8_S (I) and the synthesis and structure are reported here.

The title compound has a hetero-polycyclic structure. The 6-membering ring (C1–C6) exhibits a chair conformation. However, the other 6-membering ring (C1, C6–C10) exhibits neither a chair nor a boat conformation. The five atoms C1, C6, C8, C9 and C10 lie approximately in a plane, and the distance from C7 to the plane is 0.929 (3) Å.

The molecules of I crystalized in the *P*2_1_2_1_2_1_ space group. In the crystal structure there is an intermolecular C—H···O hydrogen-bonding interaction (Table 1), which is helpful to the stabilization of the packing. Symmetry code: *x*, 1+*y*, *z*.

### Experimental

A mixture of dihydroagarofuran (3.34 g, 10 mmol) and methanesulfonyl chloride (3.5 mL, 44 mmol) in dry Pyridine (20 mL) was stirred over night at room temperature. When the reaction was completed, 2 mL methol was added to the reaction mixture to quench the reaction, then 50 mL water was added to the mixture and it was extracted with ethyl acetate. The ethyl acetate layer was washed with 50 mL of water, 15 mL of saturated sodium chloride and dried over anhydrous sodium sulfate and was separated on a silica gel column chromatography with a gradient of petroleum ether and ethyl acetate as eluent to yield 1.87 g of the title compound. The compound was then dissolved in THF, and colorless crystals were formed on slow evaporation at room temperature over one week.

### Refinement

All H atoms were placed in geometrically calculated positions and refined using a riding model with C—H = 0.93 Å and N—H = 0.86 Å and with *U*_iso_(H) = 1.2*U*_eq_(C, N).

### Crystal data

**Table:**

C_16_H_24_O_8_S	*F*(000) = 800
*M**_r_* = 376.41	*D*_x_ = 1.472 Mg m^−^^3^
Orthorhombic, *P*2_1_2_1_2_1_	Mo *K*α radiation, λ = 0.71073 Å
Hall symbol: P 2ac 2ab	Cell parameters from 2687 reflections
*a* = 9.530 (3) Å	θ = 2.3–22.5°
*b* = 10.228 (3) Å	µ = 0.23 mm^−^^1^
*c* = 17.424 (5) Å	*T* = 296 K
*V* = 1698.3 (9) Å^3^	Block, colorless
*Z* = 4	0.25 × 0.22 × 0.21 mm

### Data collection

**Table:**

Bruker APEXII CCD diffractometer	3151 independent reflections
Radiation source: fine-focus sealed tube	2633 reflections with *I* > 2σ(*I*)
graphite	*R*_int_ = 0.034
φ and ω scans	θ_max_ = 25.5°, θ_min_ = 2.3°
Absorption correction: multi-scan (*SADABS*; Sheldrick, 1996)	*h* = −11→7
*T*_min_ = 0.944, *T*_max_ = 0.953	*k* = −12→12
8175 measured reflections	*l* = −21→20

### Refinement

**Table:**

Refinement on *F*^2^	Secondary atom site location: difference Fourier map
Least-squares matrix: full	Hydrogen site location: inferred from neighbouring sites
*R*[*F*^2^ > 2σ(*F*^2^)] = 0.039	H-atom parameters constrained
*wR*(*F*^2^) = 0.085	*w* = 1/[σ^2^(*F*_o_^2^) + (0.0384*P*)^2^ + 0.135*P*] where *P* = (*F*_o_^2^ + 2*F*_c_^2^)/3
*S* = 1.06	(Δ/σ)_max_ < 0.001
3151 reflections	Δρ_max_ = 0.19 e Å^−^^3^
232 parameters	Δρ_min_ = −0.20 e Å^−^^3^
0 restraints	Absolute structure: Flack (1983), 1325 Friedel pairs
Primary atom site location: structure-invariant direct methods	Flack parameter: 0.13 (9)

### Special details

**Table:**

Geometry. All esds (except the esd in the dihedral angle between two l.s. planes) are estimated using the full covariance matrix. The cell esds are taken into account individually in the estimation of esds in distances, angles and torsion angles; correlations between esds in cell parameters are only used when they are defined by crystal symmetry. An approximate (isotropic) treatment of cell esds is used for estimating esds involving l.s. planes.
Refinement. Refinement of F^2^ against ALL reflections. The weighted R-factor wR and goodness of fit S are based on F^2^, conventional R-factors R are based on F, with F set to zero for negative F^2^. The threshold expression of F^2^ > 2sigma(F^2^) is used only for calculating R-factors(gt) etc. and is not relevant to the choice of reflections for refinement. R-factors based on F^2^ are statistically about twice as large as those based on F, and R- factors based on ALL data will be even larger.

### Fractional atomic coordinates and isotropic or equivalent isotropic displacement parameters (Å^2^)

**Table:**

	*x*	*y*	*z*	*U*_iso_*/*U*_eq_	
C1	0.8202 (3)	0.4554 (2)	0.91912 (13)	0.0328 (6)	
C2	0.9087 (3)	0.5765 (2)	0.90120 (14)	0.0346 (6)	
H2	0.8965	0.6079	0.8485	0.042*	
C3	1.0539 (3)	0.5224 (3)	0.91630 (14)	0.0426 (7)	
H3	1.1223	0.5932	0.9220	0.051*	
C4	1.0957 (3)	0.4311 (3)	0.85095 (16)	0.0463 (7)	
H4A	1.1915	0.4021	0.8590	0.056*	
H4B	1.0930	0.4793	0.8030	0.056*	
C5	1.0002 (3)	0.3113 (3)	0.84427 (14)	0.0390 (6)	
C6	0.8443 (3)	0.3563 (2)	0.85189 (13)	0.0303 (6)	
C7	0.7308 (3)	0.2501 (3)	0.85700 (16)	0.0423 (7)	
H7	0.7108	0.2316	0.9111	0.051*	
C8	0.6076 (3)	0.3248 (3)	0.82229 (14)	0.0422 (7)	
H8	0.5317	0.2652	0.8077	0.051*	
C9	0.5590 (3)	0.4210 (3)	0.88392 (16)	0.0444 (7)	
H9	0.4683	0.4023	0.9081	0.053*	
C10	0.6664 (3)	0.4827 (2)	0.93331 (15)	0.0388 (6)	
H10	0.6393	0.4986	0.9867	0.047*	
C11	0.8932 (3)	0.4131 (3)	0.99432 (15)	0.0477 (7)	
H11A	0.8498	0.4555	1.0381	0.057*	
H11B	0.8863	0.3192	1.0010	0.057*	
C12	0.6735 (3)	0.3883 (3)	0.75151 (14)	0.0382 (6)	
C13	0.6056 (3)	0.5133 (3)	0.72187 (17)	0.0538 (8)	
H13A	0.6224	0.5830	0.7576	0.081*	
H13B	0.5063	0.5000	0.7164	0.081*	
H13C	0.6452	0.5356	0.6729	0.081*	
C14	0.6857 (3)	0.2946 (3)	0.68275 (15)	0.0531 (8)	
H14A	0.7452	0.3329	0.6444	0.080*	
H14B	0.5943	0.2793	0.6615	0.080*	
H14C	0.7254	0.2132	0.6995	0.080*	
C15	1.0454 (4)	0.2017 (3)	0.89920 (18)	0.0582 (9)	
H15A	0.9702	0.1399	0.9047	0.087*	
H15B	1.0677	0.2384	0.9484	0.087*	
H15C	1.1266	0.1582	0.8788	0.087*	
C16	0.7165 (3)	0.8404 (3)	0.88369 (19)	0.0586 (8)	
H16A	0.6393	0.8104	0.9143	0.088*	
H16B	0.7223	0.7891	0.8377	0.088*	
H16C	0.7025	0.9306	0.8705	0.088*	
O1	1.0156 (2)	0.2516 (2)	0.76988 (11)	0.0515 (5)	
H1	0.9832	0.3003	0.7370	0.077*	
O2	0.7634 (3)	0.13165 (18)	0.81877 (13)	0.0598 (6)	
H2A	0.8383	0.1396	0.7961	0.090*	
O3	1.0372 (2)	0.4519 (2)	0.98764 (10)	0.0540 (6)	
O4	0.59795 (19)	0.56021 (18)	0.87714 (11)	0.0489 (5)	
O5	0.81435 (16)	0.42256 (15)	0.77981 (8)	0.0310 (4)	
O6	0.87426 (18)	0.67549 (17)	0.95895 (9)	0.0407 (4)	
O7	0.9879 (2)	0.85121 (19)	0.88633 (12)	0.0590 (6)	
O8	0.8610 (2)	0.89333 (19)	1.00595 (11)	0.0558 (5)	
S1	0.87153 (7)	0.82442 (6)	0.93543 (4)	0.04001 (18)	

### Atomic displacement parameters (Å^2^)

**Table:**

	*U*^11^	*U*^22^	*U*^33^	*U*^12^	*U*^13^	*U*^23^
C1	0.0404 (14)	0.0302 (13)	0.0278 (13)	−0.0028 (12)	0.0013 (11)	0.0036 (11)
C2	0.0408 (15)	0.0339 (14)	0.0293 (12)	−0.0035 (12)	−0.0021 (11)	−0.0016 (12)
C3	0.0402 (16)	0.0470 (17)	0.0406 (15)	−0.0038 (14)	−0.0085 (12)	0.0057 (14)
C4	0.0324 (15)	0.0525 (19)	0.0539 (17)	0.0080 (14)	−0.0023 (13)	0.0076 (15)
C5	0.0423 (15)	0.0369 (15)	0.0378 (14)	0.0094 (14)	−0.0046 (12)	0.0021 (13)
C6	0.0372 (15)	0.0265 (13)	0.0273 (12)	0.0023 (11)	−0.0007 (11)	0.0044 (10)
C7	0.0555 (17)	0.0296 (14)	0.0418 (16)	−0.0075 (14)	0.0082 (14)	−0.0024 (13)
C8	0.0367 (15)	0.0397 (16)	0.0502 (15)	−0.0110 (14)	0.0013 (12)	−0.0043 (14)
C9	0.0360 (14)	0.0453 (17)	0.0518 (16)	−0.0074 (13)	0.0099 (13)	−0.0013 (14)
C10	0.0430 (16)	0.0378 (15)	0.0355 (13)	−0.0011 (12)	0.0092 (13)	0.0052 (13)
C11	0.0603 (19)	0.0474 (17)	0.0353 (14)	0.0002 (15)	−0.0053 (14)	0.0032 (13)
C12	0.0309 (14)	0.0440 (17)	0.0396 (15)	−0.0021 (13)	−0.0042 (12)	−0.0073 (12)
C13	0.0502 (19)	0.064 (2)	0.0476 (16)	0.0101 (16)	−0.0130 (15)	0.0021 (16)
C14	0.0508 (17)	0.062 (2)	0.0463 (17)	−0.0026 (16)	−0.0084 (14)	−0.0189 (15)
C15	0.065 (2)	0.0468 (19)	0.063 (2)	0.0182 (16)	−0.0101 (16)	0.0088 (17)
C16	0.061 (2)	0.0390 (18)	0.076 (2)	−0.0010 (16)	−0.0203 (16)	0.0003 (17)
O1	0.0583 (13)	0.0482 (13)	0.0481 (12)	0.0175 (11)	0.0052 (11)	−0.0063 (10)
O2	0.0803 (16)	0.0256 (10)	0.0734 (15)	−0.0025 (11)	0.0017 (12)	−0.0087 (10)
O3	0.0535 (13)	0.0666 (14)	0.0420 (11)	0.0020 (11)	−0.0171 (10)	0.0074 (11)
O4	0.0431 (11)	0.0423 (12)	0.0614 (12)	0.0022 (10)	0.0045 (9)	0.0029 (10)
O5	0.0323 (9)	0.0312 (9)	0.0294 (8)	−0.0006 (8)	−0.0029 (7)	0.0035 (8)
O6	0.0544 (11)	0.0357 (10)	0.0318 (9)	−0.0046 (10)	−0.0022 (8)	−0.0062 (8)
O7	0.0615 (13)	0.0448 (13)	0.0707 (13)	−0.0125 (11)	0.0216 (11)	−0.0122 (11)
O8	0.0592 (13)	0.0517 (12)	0.0565 (12)	−0.0056 (11)	−0.0002 (11)	−0.0267 (10)
S1	0.0402 (4)	0.0345 (4)	0.0453 (4)	−0.0067 (3)	0.0010 (3)	−0.0108 (3)

### Geometric parameters (Å, °)

**Table:**

C1—C10	1.512 (3)	C10—O4	1.419 (3)
C1—C2	1.531 (4)	C10—H10	0.9800
C1—C11	1.545 (3)	C11—O3	1.433 (3)
C1—C6	1.566 (3)	C11—H11A	0.9700
C2—O6	1.465 (3)	C11—H11B	0.9700
C2—C3	1.513 (4)	C12—O5	1.472 (3)
C2—H2	0.9800	C12—C13	1.523 (4)
C3—O3	1.446 (3)	C12—C14	1.538 (4)
C3—C4	1.525 (4)	C13—H13A	0.9600
C3—H3	0.9800	C13—H13B	0.9600
C4—C5	1.531 (4)	C13—H13C	0.9600
C4—H4A	0.9700	C14—H14A	0.9600
C4—H4B	0.9700	C14—H14B	0.9600
C5—O1	1.440 (3)	C14—H14C	0.9600
C5—C15	1.536 (4)	C15—H15A	0.9600
C5—C6	1.561 (3)	C15—H15B	0.9600
C6—O5	1.455 (3)	C15—H15C	0.9600
C6—C7	1.535 (4)	C16—S1	1.738 (3)
C7—O2	1.417 (3)	C16—H16A	0.9600
C7—C8	1.526 (4)	C16—H16B	0.9600
C7—H7	0.9800	C16—H16C	0.9600
C8—C9	1.528 (4)	O1—H1	0.8200
C8—C12	1.529 (4)	O2—H2A	0.8200
C8—H8	0.9800	O6—S1	1.5777 (19)
C9—O4	1.476 (3)	O7—S1	1.427 (2)
C9—C10	1.478 (4)	O8—S1	1.4201 (18)
C9—H9	0.9800		
			
C10—C1—C2	114.7 (2)	O4—C10—C9	61.24 (17)
C10—C1—C11	110.5 (2)	O4—C10—C1	115.9 (2)
C2—C1—C11	98.7 (2)	C9—C10—C1	119.8 (2)
C10—C1—C6	112.6 (2)	O4—C10—H10	116.2
C2—C1—C6	106.88 (19)	C9—C10—H10	116.2
C11—C1—C6	112.8 (2)	C1—C10—H10	116.2
O6—C2—C3	109.8 (2)	O3—C11—C1	106.6 (2)
O6—C2—C1	107.20 (19)	O3—C11—H11A	110.4
C3—C2—C1	99.9 (2)	C1—C11—H11A	110.4
O6—C2—H2	113.0	O3—C11—H11B	110.4
C3—C2—H2	113.0	C1—C11—H11B	110.4
C1—C2—H2	113.0	H11A—C11—H11B	108.6
O3—C3—C2	103.4 (2)	O5—C12—C13	107.5 (2)
O3—C3—C4	111.4 (2)	O5—C12—C8	101.85 (19)
C2—C3—C4	109.5 (2)	C13—C12—C8	117.1 (2)
O3—C3—H3	110.8	O5—C12—C14	109.9 (2)
C2—C3—H3	110.8	C13—C12—C14	106.9 (2)
C4—C3—H3	110.8	C8—C12—C14	113.3 (2)
C3—C4—C5	113.0 (2)	C12—C13—H13A	109.5
C3—C4—H4A	109.0	C12—C13—H13B	109.5
C5—C4—H4A	109.0	H13A—C13—H13B	109.5
C3—C4—H4B	109.0	C12—C13—H13C	109.5
C5—C4—H4B	109.0	H13A—C13—H13C	109.5
H4A—C4—H4B	107.8	H13B—C13—H13C	109.5
O1—C5—C4	110.3 (2)	C12—C14—H14A	109.5
O1—C5—C15	102.9 (2)	C12—C14—H14B	109.5
C4—C5—C15	111.7 (2)	H14A—C14—H14B	109.5
O1—C5—C6	107.38 (19)	C12—C14—H14C	109.5
C4—C5—C6	108.9 (2)	H14A—C14—H14C	109.5
C15—C5—C6	115.4 (2)	H14B—C14—H14C	109.5
O5—C6—C7	103.96 (19)	C5—C15—H15A	109.5
O5—C6—C5	104.51 (18)	C5—C15—H15B	109.5
C7—C6—C5	117.8 (2)	H15A—C15—H15B	109.5
O5—C6—C1	108.37 (17)	C5—C15—H15C	109.5
C7—C6—C1	108.1 (2)	H15A—C15—H15C	109.5
C5—C6—C1	113.21 (19)	H15B—C15—H15C	109.5
O2—C7—C8	114.2 (2)	S1—C16—H16A	109.5
O2—C7—C6	115.0 (2)	S1—C16—H16B	109.5
C8—C7—C6	99.5 (2)	H16A—C16—H16B	109.5
O2—C7—H7	109.2	S1—C16—H16C	109.5
C8—C7—H7	109.2	H16A—C16—H16C	109.5
C6—C7—H7	109.2	H16B—C16—H16C	109.5
C7—C8—C9	106.1 (2)	C5—O1—H1	109.5
C7—C8—C12	102.5 (2)	C7—O2—H2A	109.5
C9—C8—C12	114.7 (2)	C11—O3—C3	108.27 (19)
C7—C8—H8	111.0	C10—O4—C9	61.38 (17)
C9—C8—H8	111.0	C6—O5—C12	110.91 (17)
C12—C8—H8	111.0	C2—O6—S1	119.51 (14)
O4—C9—C10	57.38 (16)	O8—S1—O7	118.56 (12)
O4—C9—C8	119.2 (2)	O8—S1—O6	104.81 (11)
C10—C9—C8	118.3 (2)	O7—S1—O6	109.17 (11)
O4—C9—H9	116.3	O8—S1—C16	110.00 (15)
C10—C9—H9	116.3	O7—S1—C16	109.34 (14)
C8—C9—H9	116.3	O6—S1—C16	103.88 (12)
			
C10—C1—C2—O6	48.0 (3)	O2—C7—C8—C12	−77.3 (3)
C11—C1—C2—O6	−69.4 (2)	C6—C7—C8—C12	45.7 (2)
C6—C1—C2—O6	173.55 (17)	C7—C8—C9—O4	102.8 (3)
C10—C1—C2—C3	162.4 (2)	C12—C8—C9—O4	−9.5 (3)
C11—C1—C2—C3	45.1 (2)	C7—C8—C9—C10	36.4 (3)
C6—C1—C2—C3	−72.0 (2)	C12—C8—C9—C10	−76.0 (3)
O6—C2—C3—O3	67.6 (2)	C8—C9—C10—O4	108.3 (3)
C1—C2—C3—O3	−44.9 (2)	O4—C9—C10—C1	−105.0 (3)
O6—C2—C3—C4	−173.6 (2)	C8—C9—C10—C1	3.3 (4)
C1—C2—C3—C4	74.0 (2)	C2—C1—C10—O4	50.3 (3)
O3—C3—C4—C5	50.7 (3)	C11—C1—C10—O4	160.8 (2)
C2—C3—C4—C5	−63.0 (3)	C6—C1—C10—O4	−72.2 (3)
C3—C4—C5—O1	162.3 (2)	C2—C1—C10—C9	120.5 (3)
C3—C4—C5—C15	−84.0 (3)	C11—C1—C10—C9	−129.0 (3)
C3—C4—C5—C6	44.7 (3)	C6—C1—C10—C9	−2.0 (3)
O1—C5—C6—O5	−46.7 (2)	C10—C1—C11—O3	−151.7 (2)
C4—C5—C6—O5	72.7 (2)	C2—C1—C11—O3	−31.1 (3)
C15—C5—C6—O5	−160.7 (2)	C6—C1—C11—O3	81.4 (3)
O1—C5—C6—C7	68.1 (3)	C7—C8—C12—O5	−37.4 (2)
C4—C5—C6—C7	−172.5 (2)	C9—C8—C12—O5	77.1 (2)
C15—C5—C6—C7	−46.0 (3)	C7—C8—C12—C13	−154.3 (2)
O1—C5—C6—C1	−164.40 (19)	C9—C8—C12—C13	−39.8 (3)
C4—C5—C6—C1	−45.0 (3)	C7—C8—C12—C14	80.6 (2)
C15—C5—C6—C1	81.6 (3)	C9—C8—C12—C14	−165.0 (2)
C10—C1—C6—O5	73.0 (2)	C1—C11—O3—C3	3.9 (3)
C2—C1—C6—O5	−53.8 (2)	C2—C3—O3—C11	25.7 (3)
C11—C1—C6—O5	−161.2 (2)	C4—C3—O3—C11	−91.7 (3)
C10—C1—C6—C7	−39.1 (3)	C1—C10—O4—C9	111.3 (3)
C2—C1—C6—C7	−165.9 (2)	C8—C9—O4—C10	−106.7 (3)
C11—C1—C6—C7	86.7 (3)	C7—C6—O5—C12	14.6 (2)
C10—C1—C6—C5	−171.5 (2)	C5—C6—O5—C12	138.72 (19)
C2—C1—C6—C5	61.7 (2)	C1—C6—O5—C12	−100.3 (2)
C11—C1—C6—C5	−45.7 (3)	C13—C12—O5—C6	137.7 (2)
O5—C6—C7—O2	85.7 (3)	C8—C12—O5—C6	14.1 (2)
C5—C6—C7—O2	−29.4 (3)	C14—C12—O5—C6	−106.2 (2)
C1—C6—C7—O2	−159.3 (2)	C3—C2—O6—S1	107.6 (2)
O5—C6—C7—C8	−36.8 (2)	C1—C2—O6—S1	−144.83 (16)
C5—C6—C7—C8	−151.9 (2)	C2—O6—S1—O8	−169.57 (16)
C1—C6—C7—C8	78.2 (2)	C2—O6—S1—O7	−41.58 (19)
O2—C7—C8—C9	162.0 (2)	C2—O6—S1—C16	75.0 (2)
C6—C7—C8—C9	−74.9 (2)		

### Hydrogen-bond geometry (Å, °)

**Table:**

*D*—H···*A*	*D*—H	H···*A*	*D*···*A*	*D*—H···*A*
C16—H16C···O2^i^	0.96	2.32	3.218 (4)	156

Symmetry codes: (i) *x*, *y*+1, *z*.
